# A Proof of Concept for Rapid Evaluation of Urban Climate Adaptations Using Simplified Simulations over an Indian City

**DOI:** 10.1175/BAMS-D-25-0197.1

**Published:** 2026-10

**Authors:** O. Brousse, A. Martilli, A. Zonato, G. Vurro, C. Simpson, O. Kenway, P. Dasgupta, C. Heaviside

**Affiliations:** a Institute of Environmental Design and Engineering, University College London, London, United Kingdom; b CIEMAT, Madrid, Spain; c CIMA Research Foundation, Savona, Italy; d Centre for Advanced Research Computing, University College London, London, United Kingdom; e Institute of Economic Growth, Delhi, India

**Keywords:** Heat islands, Temperature, Idealized models, Mesoscale models, Adaptation, Urban meteorology

## Abstract

The 2024 Indian heat waves highlighted the vulnerability of urban populations to extreme temperatures and related health risks. Current urban climate modeling practices, aimed at informing on the expected impacts of climate-sensitive designs, often rely on complex, high-resolution simulations that are time-consuming and resource-intensive. These “real case” scenarios, while valuable, may also not explore the full range of adaptive options available to cities. We therefore propose to use simpler, more efficient modeling approaches using “idealized” scenarios to inform early stage decision-making, especially during research and design phases. We demonstrate this by running 100 simulations per common urban climate intervention—including cool roofs, urban greening, or air conditioning—using the WRF building effect parameterization coupled with its building energy model (BEP–BEM) urban climate model. Focusing on the city of Bhubaneswar, Odisha, India, we quantify how incremental one-percentage-point changes in each intervention may affect local temperatures and surface energy balance during the hottest-ever recorded day and the warmest day of 2024 following the wet monsoon period. We then compare outcomes from idealized simulations with a selection of real-case scenarios based on modeled temperature changes to see if idealized simulation outputs are reliable. We show that idealized simulations can deliver clear, actionable insights on how common urban climate interventions can impact the local climate, useful for planners and designers. Limitations from simple two-dimensional simulations are also identified suggesting that our methods should be seen as a first actionable tool for identifying viable adaptation paths that will require further three-dimensional realistic simulations to estimate the expected impacts with greater certainty.

## 1 Introduction

In the context of the changing climate and increasing urbanization globally, rising temperatures in the urban environment and its effect on health and energy consumption are a matter of serious concern. Many have proposed tackling overheating through climate-sensitive designs and interventions at the city and neighborhood scales, but the information required to assess the adequacy of these designs and interventions locally has not always been available. Rapid development and expansion of cities over recent decades have not always been matched by increased coverage of weather sensor networks to offer reliable local urban climate data that could inform decision-making (Grimmond et al. 2015). Despite optimism behind new and increasing sources of data, like personal weather stations (Chapman et al. 2017), spatial distribution of observational climate data remains unequally distributed across scales, with informal settlements and most deprived areas usually poorly covered (Brousse et al. 2024a; Creutzig et al. 2019; Van de Walle et al. 2022). Additionally, very few, if any, longitudinal studies based on long-term climate records have been performed on the impact of interventions on the local climate at neighborhood and/or city scales. This means that most of our current understanding of the expected impact from climate-sensitive designs at the city scale mainly comes from physical urban climate models (Krayenhoff et al. 2021; Santamouris et al. 2017) or statistical models trained on remotely sensed data from satellites (Srivastava et al. 2024).

Urban climate models now provide the ability to simulate a wide range of built-environment interventions, for example, several single- and multilayer urban climate models [such as Town Energy Balance (TEB) and building effect parameterization coupled with its building energy model (BEP–BEM); Martilli et al. 2002; Masson et al. 2002] now integrate green roofs or solar PV, as well as air conditioning or even street-level tree canopy into mesoscale meteorological models (such as WRF and COSMO; Chen et al. 2011; Rockel et al. 2008). Nevertheless, these models require large computational resources, so choices must be made over what scenarios to model and at what level of detail. This includes prior choices on the extent of the intervention scenario, which are often based on local policy decisions, for example, greening scenarios supported by local governments (Serras et al. 2026; Takacs et al. 2025), or on maximum deployment of the intervention to quantify the maximum possible impact and thereby maximize signal to noise (Brousse et al. 2024b). Many studies employing urban climate models perform simulations of urban climates at a high level of spatial detail (with many now using a 1-km horizontal grid), but the computational resources required for this mean that simulations are typically short. Often, a high level of spatial detail combined with a high level of physically detailed modeling of interventions means that simulation periods often cover only a single summer season or even a heat wave period of a few days to fit within resource constraints. It is also rare to repeat simulations with perturbations to test the stability of results.

Aside from the effect of better representation of physical processes such as convection when atmospheric grid sizes are smaller, spatial detail is often presented as important for making information derived from urban climate models locally relevant and making the simulations more “realistic.” As a result, some studies have prioritized apparent spatial detail while reducing physical detail, for example, driving an urban model with atmospheric data without the urban model feeding back into an atmospheric model—as in so-called offline simulations (Pappaccogli et al. 2025)—or using a nondynamic atmospheric model, as in SUEWS, Urbclim, or TARGET (Järvi et al. 2011; De Ridder et al. 2015; Broadbent et al. 2019)—thereby enabling simulations to run for any number of grid cells in parallel. A good example of this practice can be found in the study by Nice et al. (2022) which employed the microscale nondynamic atmospheric VTUF-3D model (Nice et al. 2018) to run ~ 10k offline simulations. Some studies have presented simulated urban near-surface air temperature with horizontal resolution of 100 m; however, intuitively, spatial patterns at smaller scales should be more uncertain and less robust. Producing information at finer spatial scales therefore also presents a trade-off against other kinds of model detail (usually physical detail).

The effectiveness of interventions is estimated by comparing the output (usually near-surface air temperature) of different runs, but uncertainties are difficult to quantify. Usually, the baseline scenario simulation is compared to air temperature observations to provide validation and an estimate of error. Krayenhoff et al. (2021) suggested that the estimated impacts of interventions should only be considered valid if the impact on the local climate is greater than the error between the baseline simulation and the observations—a condition that is rarely met. Urban climate model runs often have mean absolute errors against observations on the order of 1°C, and this is widely treated as an acceptable level of agreement. Achieving a high level of agreement requires tuning the input of the baseline scenario, and computational resources often limit this. However, the error of the baseline run against observations is not the same as the error on the intervention effectiveness; generally, there are no relevant observations against which to compare intervention scenarios because they are counterfactual. We may also have higher confidence that (e.g.,) increased albedo will decrease temperature than in the absolute predictions of temperature because some sources of error may cancel, or if increased albedo consistently decreases temperature across many simulations. Usually, the validity of urban climate models is established through case studies where flux tower or other detailed observations have been collected, for example, through the BUBBLE project (Rotach et al. 2005; Salamanca and Martilli 2010). But, it is not usually practical to propagate this forward into an error estimate for any given scenario.

Another widely used approach is the use of statistical models based on dense networks of air temperature measurements (e.g., Taylor et al. 2024) or surface information derived from satellite instruments, like greenness (Wu et al. 2025) or surface temperatures (Iungman et al. 2023)—the latter having their own challenges in terms of representativeness of the actual thermal environment people occupy (Zhan et al. 2025). A major issue related to the employment of such models is that they represent changes to the urban thermal environment based on spatial comparisons between environments inside and/or outside the cities’ boundaries (e.g., reduction in air temperature linked to greater NDVI values observed in rural areas), rather than interventions on the urban environment. This means that such models lack interpretability by being extrapolated based on unseen conditions, thus also questioning their external validity. In addition, the modeled relationship is often considered linear, while little is known about the (non)linearity of the expected climate impact in dynamic atmospheric environments by local interventions and the extension of their deployment (e.g., Takacs et al. 2025 or Taylor et al. 2024). Nevertheless, despite these limitations, statistical approaches offer considerable utility: They enable rapid examination of extensive spatial trends, require minimal computational resources, and can produce initial hypotheses about which surface characteristics might influence local weather. Their efficiency makes them suitable for scoping exercises, especially where fully resolved urban climate simulations remain computationally demanding or impractical.

That said, these advantages do not replace the need for physics-based models (i.e., urban climate models), which deal with the governing equations of energy, momentum, and mass fluxes within the urban canopy layer, thereby upholding interpretability, causality, and technical fidelity. This provides robustness under unforeseen or unprecedented circumstances—including extreme heat events, altered soil moisture (SM) dynamics, or combinations of different interventions. Diverging from empirical models, physics-based models can simulate counterfactual modifications to urban morphology (e.g., building height or road width), materials (e.g., tiled or asphalted roofs), vegetation (e.g., grassland or deciduous tree forests), and anthropogenic emissions (e.g., exhaust hot air from air conditioning or fumes from industries and combustion vehicles), while reliably capturing nonlinear processes such as heat storage and release (e.g., heat trapped in the soil or building materials), aerodynamic coupling (e.g., the impact of buildings on the atmospheric circulation), or drought-stressed evapotranspiration (e.g., when lack of water limits how much moisture can be released by watered surfaces and/or vegetation). Consequently, they enable rigorous experimental control, yield externally valid insights, and support extrapolation to future climates in ways that purely data-driven models cannot achieve on their own.

In the face of these modeling challenges, we argue that simplified urban climate models offer an alternative to both these standard modeling approaches. Ultimately, the aim of many urban climate modeling studies is to understand the effectiveness of various interventions in improving thermal comfort and/or protecting against extremes of temperature. In such studies, we argue that it is productive to make spatial representation of the city more abstract to enable exploration of a wider range of scenarios and weather conditions without compromising on the physical detail of the urban climate model. Much of the computational complexity of urban climate models comes from representing the atmospheric fluid dynamics in three-dimensional space across a large number of grid cells. We propose that urban climate models with two-dimensional atmospheric dynamics [i.e., with only one horizontal dimension, similar to URBMET from Bornstein (1975)], that still resolve the detailed interaction of urban canyons with the atmosphere and integrate climate adaptation models, could support local tailoring of urban climate designs and interventions. Such models would offer a quick and efficient tool for quantifying the impact of common climate adaptation/mitigation interventions and urban climate-sensitive designs under multiple deployment scales. Because simplified models can be run through a wider range of scenarios than traditional urban climate models, they can be used to find suitable deployment strategies in line with local conditions. This could mean, for example, a series of simulations where green space is increased incrementally through a wide range of values or a series of simulations that tests interventions individually as well as in combination. When the simpler models use the same urban canopy parameterization as the more complex models, they can be used to screen for effective interventions prior to running a more computationally expensive three-dimensional model. If widely adopted, such preliminary analyses could also advance urban climate science by increasing the understanding of how local climate effects scale with deployment under differing background climatic conditions.

In this paper, we present the outcomes from the first idealized urban climate model scenario for WRF BEP–BEM to study the impact of key interventions on urban climate. Our study focuses on the city of Bhubaneshwar in the east of India, as recent extreme heat waves in Indian cities call for urgent climate action and local urban climate interventions (Dasgupta et al. 2024). India has seen its urban population rising in the late twentieth century and has observed uncontrolled development of informal settlements. In line with the characteristic urban climate data scarcity that exists globally (Lizana et al. 2023), urban climate observations in India remain sparse and often lack valuable information on the potential of climate interventions. Currently, most of the evidence provided on the effect of urban climate interventions comes from modeling case studies of varying complexities that are either limited by the amount of simulation days and/or the set of studied urban climate interventions (e.g., Borah et al. 2026; Khorat et al. 2025; Khan et al. 2026) and which, again, come at great computational costs. In the face of these challenges and the urgency to resolve them, we argue that such simple urban climate models could be central to offering valuable information to shape Indian cities. It is indeed paramount to offer tools that can rapidly inform local decision-makers about viable urban climate interventions and support action implementation, as noted by Mehryar et al. (2022), while also enabling discussion of their costs and benefits under determined local environmental conditions that will foster transformative capacities. Similar to the CRAFT tool developed for estimating the impact of climate change mitigation strategies on health (Symonds et al. 2020), we present details of a simplified, idealized modeling framework designed to rapidly respond to current urban climate challenges. We argue that our modeling strategy enables quick, approximate quantification of how multiple urban climate interventions affect the local climate, illustrated through air temperatures and surface energy balance components that can help support preliminary research directions and policy discussions before committing resources to comprehensive analysis. Once promising interventions are identified for a specific city, more complex models can provide the detailed simulations needed for implementation. We demonstrate the latter approach by examining how well outputs from idealized simulations transfer to more realistic scenarios. Our framework aims to clarify the potential magnitude of impact that climate-sensitive urban design interventions might achieve.

## 2 Methods

To perform our study, prior model development was required to run the WRF Model (Chen et al. 2011) in idealized mode with a two-dimensional representation of a city and with the BEP–BEM (Martilli et al. 2002; Salamanca et al. 2010; Salamanca and Martilli 2010). We chose to run the BEP–BEM as this complex urban climate model offers many intervention schemes, such as street trees, street-level green areas, air conditioning, roof solar photovoltaic panels, and changes to radiative and thermal parameters at the roof/wall/street level—allowing the representation, for example, of cool roofs. However, some of these intervention schemes did not offer fractional changes to test the impact of small changes in their deployment, like air conditioning, which required further model development. Last, a specific modeling strategy had to be set up to provide input parameters to the idealized model.

**Fig. 1:**
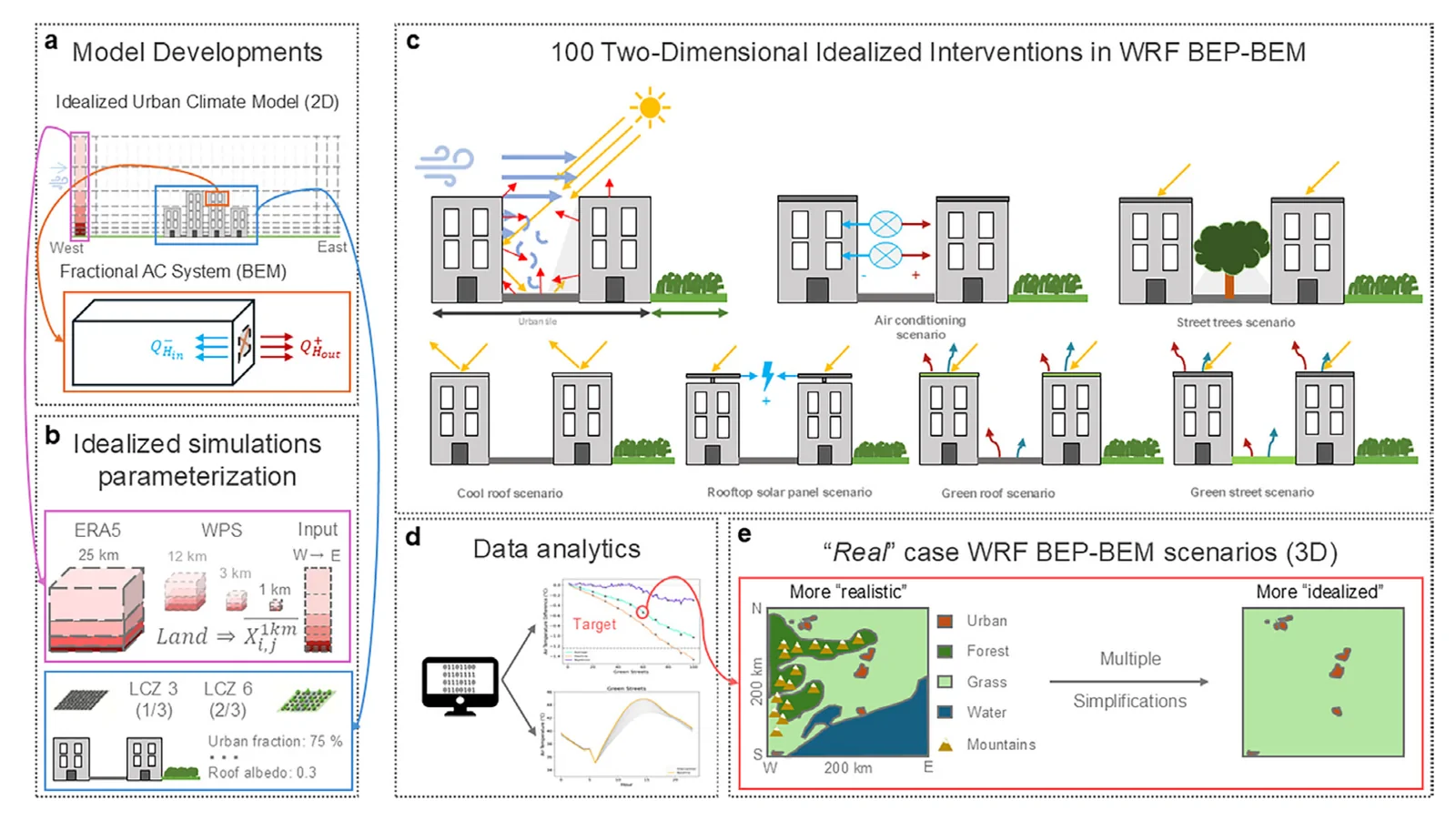
Framework of the methods employed in the manuscript. (a) Before running the idealized simulations, some model developments were necessary to represent the impact of urban areas on the local climate in two dimensions (*X* and *Z*) and in fractional deployments of AC systems. (b) Inputs for the vertical atmospheric columns and initial surface conditions are then derived from ERA5 and LCZs. (c) This then drives 100 simulations employing WRF BEP–BEM as an urban climate model to test the impact of multiple climate adaptive interventions on the urban climate of Bhubaneshwar. (d) Subsequent data analysis looking at the modeled cooling efficiency of different interventions and related impact on the surface energy balance then helps defining target temperature reductions and preferred climate sensitive designs. (e) These can then be run in real-case scenarios to look at the impact in more realistic conditions—sensitivity to more idealized initial conditions was tested.

Below, we first detail recent model developments including the idealized urban simulations and the inclusion of a fractional change in air-conditioning deployment in WRF BEP–BEM. Second, we provide information on the modeling area and model parameterization we used. Finally, we provide an exhaustive description of the different interventions that were modeled. Figure 1 provides a summary of the framework adopted in this paper.

### a Model development

The idealized modeling framework used in this study builds upon the philosophy of previous idealized two-dimensional urban simulations (Pappaccogli et al. 2020; Zonato et al. 2021), which offer an efficient and controlled environment for exploring the thermodynamic response of the urban boundary layer to specific drivers. Running WRF in a 2D configuration drastically reduces the computational burden while retaining the essential dynamical processes that shape the diurnal cycle of urban temperatures. This efficiency allows us to perform large ensembles of experiments and to systematically examine a wide range of urban geometries and air-conditioning deployment scenarios.

To enable this configuration, several modifications were introduced into WRF so that the model evolves only in the longitudinal and vertical directions, while suppressing gradients along the transverse axis (Fig. 1a). The domain is defined as a narrow two-gridcell strip aligned west–east, with periodic lateral boundary conditions applied in both directions. The two-dimensional model configuration is numerically stable for integration periods of several days, provided that wind speeds remain above a few meters per second and that vertical resolution is sufficient in the lowest atmospheric layers. However, because the periodic lateral boundaries continually recycle air masses, long integrations tend to accumulate biases in momentum and radiative fields. For this reason, and consistent with earlier studies, we recommend limiting the duration of idealized experiments to approximately 72 h. Within this window, the model reliably captures the diurnal evolution of the urban boundary layer and the sensitivity of near-surface temperature to changes in building geometry and air-conditioning deployment. Information on the initial conditions is provided in the subsequent subsection.

In addition, WRF BEP–BEM required further model development to represent the impact on air temperature and heat fluxes from fractional changes in air-conditioning deployment (Fig. 1a). As per the model development of BEM by Salamanca et al. (2010) and Salamanca and Martilli (2010), air-conditioning activation was considered to be a Boolean condition set as ON or OFF. Some studies already noted the need to include fractional changes in air-conditioning coverage as not all buildings contain air-conditioning (AC) units, leading to overestimations of the impact of AC on anthropogenic heat fluxes and thereby air temperature (Takane et al. 2024).

To do this, we considered the outgoing heat fluxes to be dependent on the fraction of the buildings covered by AC (AC_FRC_) and the floor area that is cooled by the AC units (COOL_FRC_). The outdoor and indoor temperatures estimated at each 5-m building level are then adapted accordingly to reflect the transfer of heat from the indoor environment to the outdoor environment to maintain a target temperature indoors. We therefore adapted the calculation of outgoing sensible heat fluxes, which drives the maintenance of indoor temperatures within a chosen comfort range, and given in Eqs. (19) to (22) in Salamanca et al. (2010) as

$$Q_{H_{\text{out}\;}}=\left(\frac{\left|Q_{H_{\text{need}\;}}\right|+\left|Q_{E_{\text{need}\;}}\right|}{\text{COP}}+Q_{H_{\text{need}\;}}\right)\times {\text{AC}}_{\text{FRC}}\times {\text{COOL}}_{\text{FRC}},$$

where $Q_{H_{\text{out}\;}}$ represents the outgoing sensible heat flux, $Q_{H_{\text{need}\;}}Q_{E_{\text{need}\;}}$ represents the sensible and latent heat removal needs, respectively, to reach the target temperature, and COP represents the coefficient of performance from the AC units [here left in its default value of 3.5 as per Salamanca and Martilli (2010)]. Subsequently, the indoor temperature of the indoor building at each level is adapted at each time step *t* following Eqs. (20) and (22) in Salamanca et al. (2010):

$$T_{t+1}=T_{t}+\frac{dt}{Q_{b}}\times \left(Q_{H_{t}}-Q_{H_{\text{out}\;,t}}\times {\text{AC}}_{\text{FRC}}\times {\text{COOL}}_{\text{FRC}}\right),$$

where *T*_*t*_ is the indoor temperature at each building level at time *t, Q*_*b*_ is the overall heat capacity of the indoor air, $Q_{H_{t}}$ is the current sensible heat flux generated inside the room at the given building level, and *dt* is the time step in seconds.

Note that this is intended to estimate the average heat fluxes into the urban environment and may not necessarily produce realistic indoor temperatures for individual buildings. The latter could potentially induce overestimated cooling requirements in certain buildings and consequently lead to overestimated releases of anthropogenic heat fluxes in the atmosphere. However, the purpose of the AC model in BEP–BEM is not to study indoor temperatures of individual buildings but rather to estimate the impact of homogeneous AC deployments across the built environment.

### b Study area and model parameterization

To demonstrate the potential application of idealized simulations for guiding decision-making in places that require rapid adaptation to climate changes, we chose to focus on the city of Bhubaneshwar in the state of Odisha, India, for an extremely warm day of the year 2024. The rationale for this choice builds on an ongoing study in Bhubaneswar that seeks to develop interventions to reduce heat stress among vulnerable populations in the city (see https://chhanw.com/ for more information on the research project; Dasgupta et al. 2024; Dasgupta et al. 2025). Below, we provide more detail on the case study geography and past climatology which supported its use for our pilot study. We also provide step-by-step explanations of choices that were made in the parameterization of the idealized simulation based on the CHHANW project objectives.

During 2024, unprecedented extreme temperatures were observed across India, with daily average temperatures above 30°C and relative humidity levels greater than 70% in the city of Bhubaneshwar (Dasgupta et al. 2024; Karrevula et al. 2025; Ravindra et al. 2024). Although heat action plans exist in India (PIB Delhi 2023; Singh et al. 2024), recent dangerous heat levels and future projections of increasing temperatures call for immediate adaptation of Indian cities (Dasgupta et al. 2024; Norgate et al. 2024). In this study, we chose to build up a city archetype that could be employed to study its reaction to different interventions under a variety of climatic conditions characteristic of Indian climates. For the purpose of this study, we constrain our analysis to the local climate in the city of Bhubaneshwar to showcase how idealized simulations could guide decision-making to meet the need for assessing various designs in this urban context. Bhubaneshwar is the capital city of Odisha and the home of ~ 837k inhabitants, or 1.1 million for the metropolitan area, according to the 2011 census (Census Organization of India 2011). In 2011, 92 Indian cities had more than 500k inhabitants with 10 of them in the range of 800k–900k inhabitants, similar to Bhubaneshwar, and 70 in the range of 500k–1.5M inhabitants. This further demonstrates how Bhubaneshwar can be considered as a valid typical Indian regional metropolis, suitable for our case study.

To run two-dimensional idealized simulations, initial conditions describing the land-use and land-cover physical and thermal parameters are necessary, as well as information on the atmospheric column at all model levels. Obtaining information on the atmospheric temperatures at different heights relies on the deployment of weather balloons that record common weather variables throughout the troposphere. Such data are sparsely available across the globe, and no records were obtained around Bhubaneshwar. Therefore, instead of relying on local observations, we decided to rely on the globally available ERA5 climate reanalysis at 0.25° horizontal resolution (Hersbach et al. 2020; Fig. 1b). To do this, we first used the WRF preprocessing system (WPS) to obtain model-ready initial conditions for a “real” three-dimensional case scenario across a regional domain of 200 km × 200 km at a 1-km horizontal resolution centered over the city of Bhubaneshwar. The pressure at the top of the model is set at 2000 hPa and subdivided into 60 vertical levels, scaling pressure levels from the surface to model top. We then averaged the input values for the real-case scenario for potential air temperature and water vapor at each model level heights. Westerly wind is set at 5 m s^− 1^, constant with height. Surface temperature is set according to the selected dry season at 308 K. The specific humidity is set at 25.56 g kg^− 1^ close to the surface and decreases linearly to the model top. Soil moisture is set at 0.25 m^3^ m^− 3^ based on averages obtained from ERA5 for land pixels. This way, we obtained an average atmospheric column, valid for the dry season of our region of interest around Bhubaneshwar. This information can then be interpolated to the chosen model vertical layers using the idealized simulations preprocessing system. Microphysics and cumulus schemes are disabled to avoid cloud formation, while horizontal turbulent diffusion coefficients remain constant at 300 m^2^ s^− 1^.

The atmospheric column was parameterized at 1200 UTC, or 1730 IST 28 May, after identifying extremely warm and dry days over Bhubaneshwar using daily average temperatures, soil moisture levels, and daily total precipitation for land pixels only given by the land–sea mask from ERA5. We considered a 12-h spinup time in our simulations, with a time step of 5 s, and focused our analysis on the subsequent 48 h that cover the hottest day of May 2024 and span from 0600 IST 29 May to 0600 IST 31 May, exclusive of the last hour.

For the land-use land-cover information, we chose to characterize our city archetype in the form of a weighted composite of characteristic residential local climate zones (LCZs) that compose Indian cities. According to the global LCZ map by Demuzere et al. (2022b), cities of the ecoregion “tropical, subtropical forest in Asia”—mostly composed of Indian cities in the global LCZ map training sample—are predominantly composed of low-rise residential areas. We therefore chose to consider 2/3 of the land cover as LCZ 6 and 1/3 as LCZ 3 (Fig. 1b), to still reflect the high density of populated Indian cities. We then weighted based on their proportion in coverage the thermal, radiative, and the morphological parameters from LCZs 3 and 6, as given by the WUDAPT-TO-WRF strategy (Brousse et al. 2016; Demuzere et al. 2022a). This translated in an archetype city with a built-up surface fraction of 75%, a building width of 10 m, a building height of 6.25 m, and a street width of 18.8 m (see S1 in the online supplemental material for more detailed information). It is important to note that thermal and radiative parameters applied in these simulations are not specific to Indian cities, which could influence model outputs (Van de Walle et al. 2021). Bhubaneshwar exhibited an urban extension of ~20 km in all directions, justifying a city extension of 20 km (20 grid cells of 1 km), which we located in the center of our two-dimensional idealized domain of 200 km to allow land–atmosphere interactions to take place upwind and have sufficient dispersion downwind.

The domain is therefore defined as a narrow two-gridcell strip aligned west–east, with periodic lateral boundary conditions applied in both directions (Figs. 1a,b). In the center of the domain, our idealized “city” is positioned on completely flat terrain, represented as a single column of urban grid cells surrounded by homogeneous rural land characterized by grasslands, typical of the surroundings of Bhubaneshwar. Grasslands are based on the USGS land-use categories, no orography is set up in the surroundings, and the idealized simulations are set at a latitude of 20°, which is the latitude of Bhubaneswar. The numerical grid consists of 200 horizontal cells with a resolution of 1 km and 55 vertical levels refined close to the ground to capture street-canyon processes and the evolution of the nocturnal boundary layer. The model top is set at a height of 16 km with 5000 m of additional damping.

On this set up, we ran the WRF Model, version 4.5, with the Dudhia shortwave and RRTM longwave schemes (Dudhia 1989; Mlawer et al. 1997). We used the Bougeault–Lacarrere planetary boundary layer parameterization (Bougeault and Lacarrere 1989), valid with WRF BEP–BEM (Fig. 1c). We employed the revised MM5 Monin–Obukhov surface layer scheme (Jiménez et al. 2012) while we chose to use the Noah-MP land surface scheme over four soil layers in its default parameterizations (Niu et al. 2011; Yang et al. 2011). Bug-fix changes from WRF v4.7 were applied to employ Noah-MP in version 4.5 with LCZ data as inputs (see related GitHub commit).

All these parameters are left unchanged across all idealized simulations that we ran for all interventions and for our baseline scenario, which we hereafter refer to as Control. To test the viability of idealized simulations for other conditions experienced in Bhubaneshwar, we repeated this exercise for 2 days identified as exceptionally warm days following the rainiest event of 2024. We followed the same procedure for the parameterization of initial conditions, but for the time period from 0600 IST 1 October to 0600 IST 3 October, with 12-h spinup time. The surface temperature was set at 302 K instead, the specific humidity at 22.81 g kg^− 1^, and the soil moisture at 0.38 m3 m^− 3^. Below, we detail each intervention scenario.

### c Idealized simulations of urban climate interventions.

To inform future decision-making on potential adaptation strategies that may be worth exploring and deploying, we decided to run each intervention scenario with an incremental deployment of 1%, summing up to 100 simulations per intervention. For cool roofs, as WRF BEP–BEM does not allow for tiling of roof surfaces, we increased the reflective capacity by 1% instead, i.e., 0.01 albedo increase per incremental step. This allowed us to explore how much of an increment in *x* intervention would be required before, for example, reaching a target of *n* degrees temperature reduction—which could also be extended to looking at how much deployment of an intervention would be necessary to achieve a similar cooling by means of actions on the density of the urban area. In addition, ranking between different interventions can be analyzed to know if certain interventions systematically perform better than others (e.g., in cooling capacity) or if, passed a certain deployment ratio, others become more interesting. Furthermore, it allowed us to see the relationships that exist between temperature reduction and extensions at which interventions are deployed in the climate of Bhubaneshwar. For example, we were able to observe the magnitude of linear and/or nonlinear impacts on temperature reduction depending on the type of interventions for both dry and wet conditions; in other words, the (non)linear sensitivity of the dynamic model to changes in the level of deployment of one intervention. This is important for urban design decision-making as it would prevent wrong assumptions on the expected cooling that would be obtained between two idealized deployments, e.g., projecting cooling capacity of a deployment of 50% green roof coverage from simulations with a deployment at 20% and 100%. Last, our proposed framework also allows decision-makers and researchers to examine the composition of the effects that underlie weather variable changes. For example, surface energy balance components can easily be analyzed alongside air temperature to investigate how each intervention affects the surface energy balance and how such changes could lead to unforeseen negative or positive impacts (e.g., through rising latent heat emissions).

We defined a set of nine interventions (Fig. 1c), where urban canyon morphologies are constant and defined as per the weighted average of LCZs 3 and 6 explained above, and where anthropogenic fluxes are computed by the BEM coupled to BEP and scaled depending on the urban fraction. Our set of idealized simulations was defined by the available intervention schemes existing in WRF BEP–BEM and included

1) a variation of urban density by modifying the urban fraction and therefore the space covered by the urban tile in each 1-km pixel divided into an urban tile and a natural tile; not to be confused with testing the impact of urbanization—the Urban Fraction scenario;
2) an activation of the air-conditioning system using Salamanca et al. (2010) scheme, and an increase in the fraction of AC system covering the buildings—the AC System scenario;
3) an increase in reflective capacity of roofs by changing the roof albedo—the Cool Roofs scenario;
4) an increase in the roofs surface covered by green roofs and an activation of the green roof scheme from Zonato et al. (2021) with grass cover and no irrigation—the Green Roofs scenario;
an increase in the street surface covered by green areas similarly represented as street-level green roofs from Zonato et al. (2021) scheme with grass cover and no irrigation—the Green Streets scenario;
5) an increase in roof solar photovoltaic panels coverage using Zonato et al. (2021) scheme—the Solar PV scenario;
6) an increase in street-level tree coverage with a leaf area index of three and a tree crown located at the second model level (5–10 m), represented in the BEP-Tree scheme (Krayenhoff et al. 2020)—the Street Trees scenario;
7) an increase in green roofs similar to the Green Roofs scenario, but also with 100% coverage of solar photovoltaic panels (100% Solar PV)—the Green Roofs PV scenario; and
8) an increase in solar photovoltaic panels similar to the Solar PV scenario, but with 100% of green roofs (100% Green Roofs)—the Solar PV GR scenario.

Once the full set of 100 simulations were run, we tested the transferability of the information obtained through our idealized simulations to “real case” scenarios. First, we investigated the model’s capability to adequately represent the ranking of cooling capacities for each intervention in idealized simulation; meaning which intervention cools better or worse than others. To test this, we ran a set of real-case simulations, where the urban climate model parameterization remained identical between the idealized and the real-case simulations. However, adaptation in the atmospheric layers needed to be made. The model height (16 km) was translated into pressure level at the top of the model and resulting in a top pressure level of 118 hPa, subdivided into 55 vertical levels. Once this was achieved, we applied a deployment level of 60% for each intervention and ran the model in a default two-way nested real-case scenario for the dry season case.

Second, we ran a subset of real-case scenarios for two chosen interventions based on their cooling capacity for both the dry and wet season cases (Figs. 1d,e). For the chosen cooling capacity and interventions, we ran a set of five real-case scenarios that leaned toward more idealized conditions to test how this would affect the similarity of the outcomes between idealized and realistic conditions (Fig. 1e). We therefore iteratively ran the model: (i) in a default two-way nested real-case scenario, (ii) with the inner domain ran in a one-way nesting setting (ON), (iii) with a flat terrain and no sea (ONFNS), (iv) with all rural land converted as grass land (ONFNSG), and last (v) with a uniform soil moisture similar to the idealized simulations (ONFNSGSM).

## 3 Results: Proof of concept on the potential of idealized simulations

**Fig. 2:**
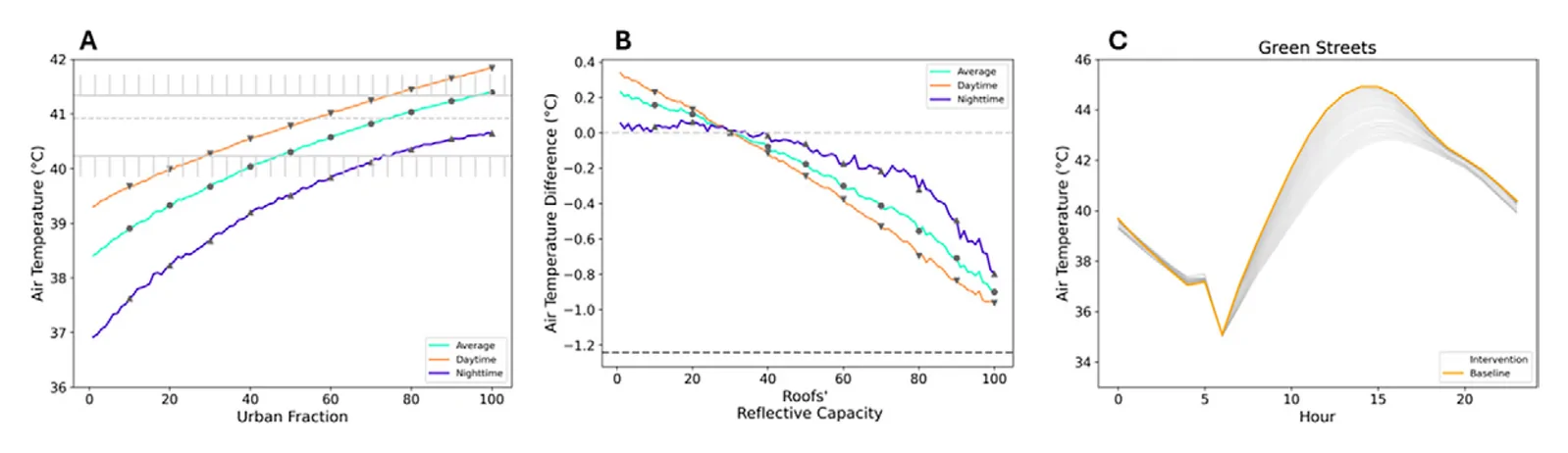
In (a), average air temperatures observed through 100 simulations show a steeper increase in temperature for each additional percentage point increase at low urban fractions. Dots represent the average temperature, upward triangles represent the nighttime average temperature (when net solar radiation is null), and upward triangles represent the daytime (when net solar radiation is positive) average temperature for multiples of 10%. Gray dotted line shows the average temperature at an urban fraction of 75%, also defining the Control scenario. Hashed lines represent the respective daytime and nighttime average temperatures of the Control scenario. Panel (b) shows a marked nighttime temperature reduction when roof albedo is above 0.80 when calculating the Cool Roofs scenario differences to the Control scenario. Zero change and maximum temperature reduction observed across all interventions are given in light gray and dark gray dotted lines, respectively. Last, panel (c) shows that when deploying Green Streets (gray lines), lower temperatures compared to the Control run (orange line) are expected across the whole day, except in nighttime hours before sunrise. Average diurnal cycle of air temperature calculated across the two simulation days, and each gray line represents a single simulation of the set of 100 simulations run for interventions.

Using the outcomes of our set of 100 simulations for each intervention, several informative patterns emerge. First, we show that simple simulations quickly inform us about the (non)linear sensitivity of the model to changes in the scale of deployment of urban climate interventions (see examples of varying urban fraction and roof reflective capacities in Figs. 2a and 2b, respectively). For example, during the dry season, we show through the Urban Fraction scenario that, while a city covering 1% of the land surface would be cooler by −2.51°C than the Control scenario (which has 75% urban fraction), and a city covering 100% of the land surface would be warmer by 0.47°C, a city covering 50% would be cooler by −0.62°C. The temperature difference between a 0% urban and 50% urban is greater than between 50% urban and 100% urban. In other words, for similar ranges of increase in urban fraction, different linear slopes obtained from least square regressions are observed with values of 0.038°C per percentage point increase from 1% to 50%, and 0.022°C from 51% to 100%, respectively. From a decision-making perspective, this could be interpreted as increasing the surface covered by small openly built cities leading to greater temperature increases than further expanding openly built cities in the same area (of 1 km in this case). Another way of looking at this example would be to say that, by reducing the urban fraction by 25% from its original 75%, daily average temperatures that are equivalent to average nighttime temperatures during extremely hot days in the current climate of Bhubaneshwar would be reached (Fig. 2a). Besides, average temperature would reach average daytime levels if the city would densify to levels where no natural areas would be left—or, 100% urban fraction. Here, we refer to daytime and nighttime temperatures as average temperatures with and without incoming solar radiation, respectively.

Regarding the effects of interventions during the warmest day experienced in Bhubaneshwar (dry season), we can see in Fig. 2b that 100% Cool Roofs would result in a maximum average cooling of 0.9°C compared to the control run which had a reflective capacity of 30%. The overall greatest average cooling across all interventions was −1.24°C brought by 100% Street Trees, a change equivalent to reducing the urban fraction to 30%. On top of this, we can see that the cooling caused by Cool Roofs, which follows a nonlinear trend. It increases faster past 80% for average nighttime cooling with a slope of −0.018°C per percentage point, compared to −0.011°C from 30% to 79%—the Control run having a roof reflectivity set at 30%. Such nonlinear trends in the average temperatures observed across all days, nighttime, or daytime hours can be better understood by looking at the average diurnal cycles of each intervention. In Fig. 2c, for example, we can see that most of the cooling brought by Green Streets occurs during the day, while at night this intervention could even cause warming. This means that although Green Streets achieves greater average cooling than Cool Roofs, with a maximum average cooling of 1.03°C compared to 0.9°C, for example, Cool Roofs would remain a better option for reducing nighttime temperatures, especially if deployed with a reflective capacity above 80%.

**Fig. 3:**
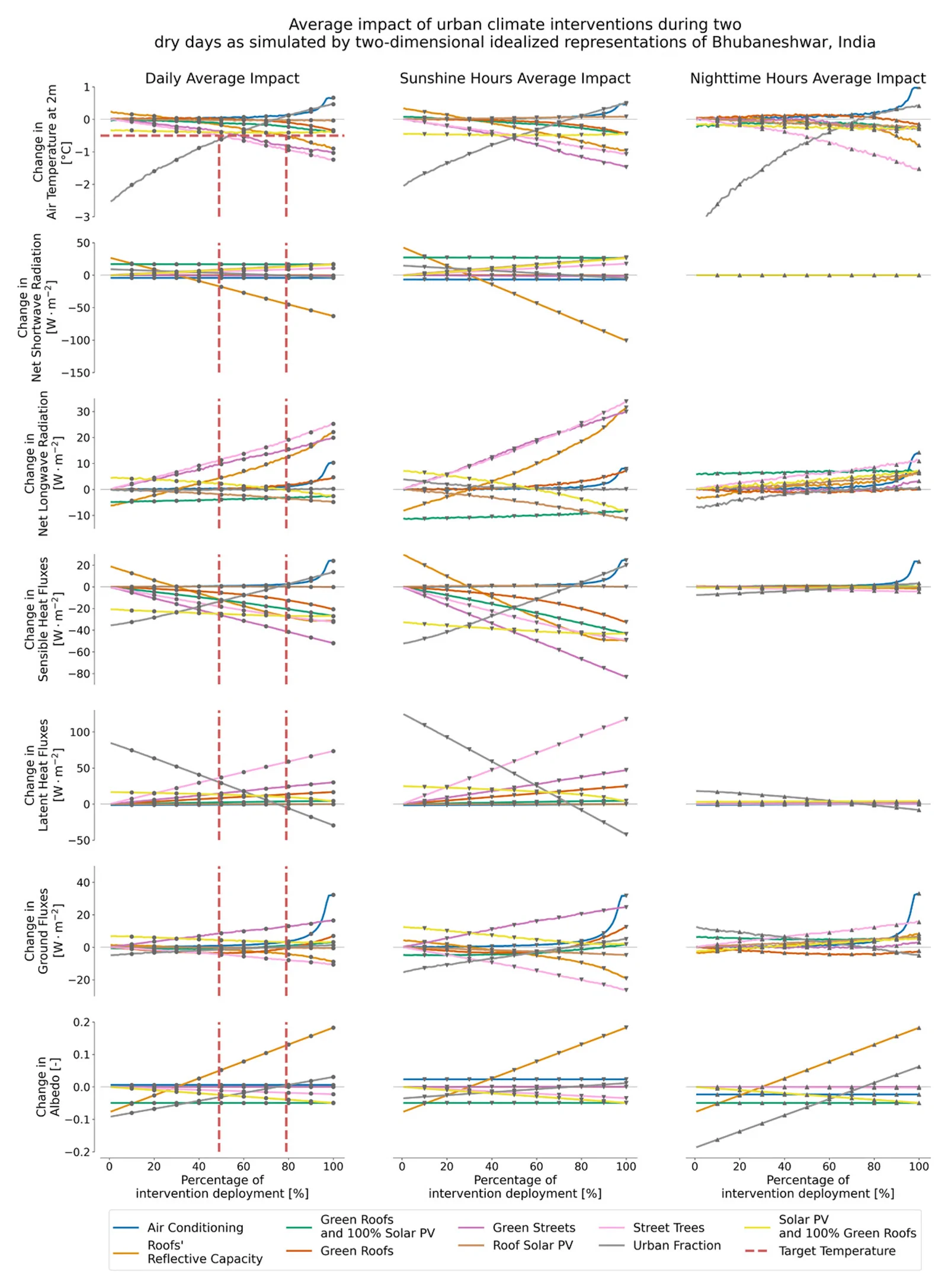
Changes in air temperature and surface energy balance components for each percentage point of change in each intervention during extremely warm days of the dry season show that the nonlinearity of the impact is not simply observed in air temperatures but across the surface energy balance components. Symbology is similar to Fig. 2b, but whole-day averages, daytime averages, and nighttime averages are split between three columns. Each line’s color represents one type of intervention. Horizontal red lines represent an idealized target for reduction of temperature set at −0.5°C for two interventions of choice (Cool Roofs and Street Trees), while vertical lines represent the resulting *x*-axis coordinate at which other differences to the surface energy balance can be read. Supplemental figures covering wet season warm days and single figures of air temperature changes per interventions are provided in the S2 (Figs. S1–S19).

Such patterns can be particularly useful during early phases of research development or decision-making. In Fig. 3, we show how by setting a temperature reduction target at −0.5°C during the warmest days of the dry season, for example, any user could look at expected changes in average air temperature at different times of day for different interventions. In addition, related changes in surface energy balance components can be easily examined and aid interpreting how the model is representing the impact of the intervention which translates into changes of air temperatures. For example, if targeting an average cooling of −0.5°C by means of Cool Roofs or Street Trees, reflective capacity should be set between 78% and 79%, while trees should cover between 49% and 50%. During the wet season, lower targets should be set as cooling capacities are lower overall. Only Street Trees reach a cooling capacity of −0.5°C at extents comprised between 70% and 71%. Therefore, when setting a target of −0.3°C for the wet season days, Cool Roofs’ reflective capacity should be set between 90% and 91%, while Street Trees should cover between 47% and 48% of the streets, similar to extents during the dry season.

Looking at the expected changes in energy balance from the target intervention deployment during the dry season days (Fig. 3), we find that while such a Cool Roofs intervention would decrease the average net solar radiation by −44.25 W m^2^, Street Trees would increase it by 5.49 W m^2^ for similar reductions in temperatures, potentially due to the observed reduction of −0.01 in urban albedo. Additionally, while Cool Roofs do not impact latent heat flux, Street Trees would increase it by 36.25 W m^2^ on average due to evapotranspiration, which could lead to increases in relative humidity and therefore additional heat stress for similar temperature reduction (Cramer et al. 2022; Simpson et al. 2023)—the latter depending on the baseline humidity and the change of humidity related to the changes in latent heat fluxes.

**Fig. 4:**
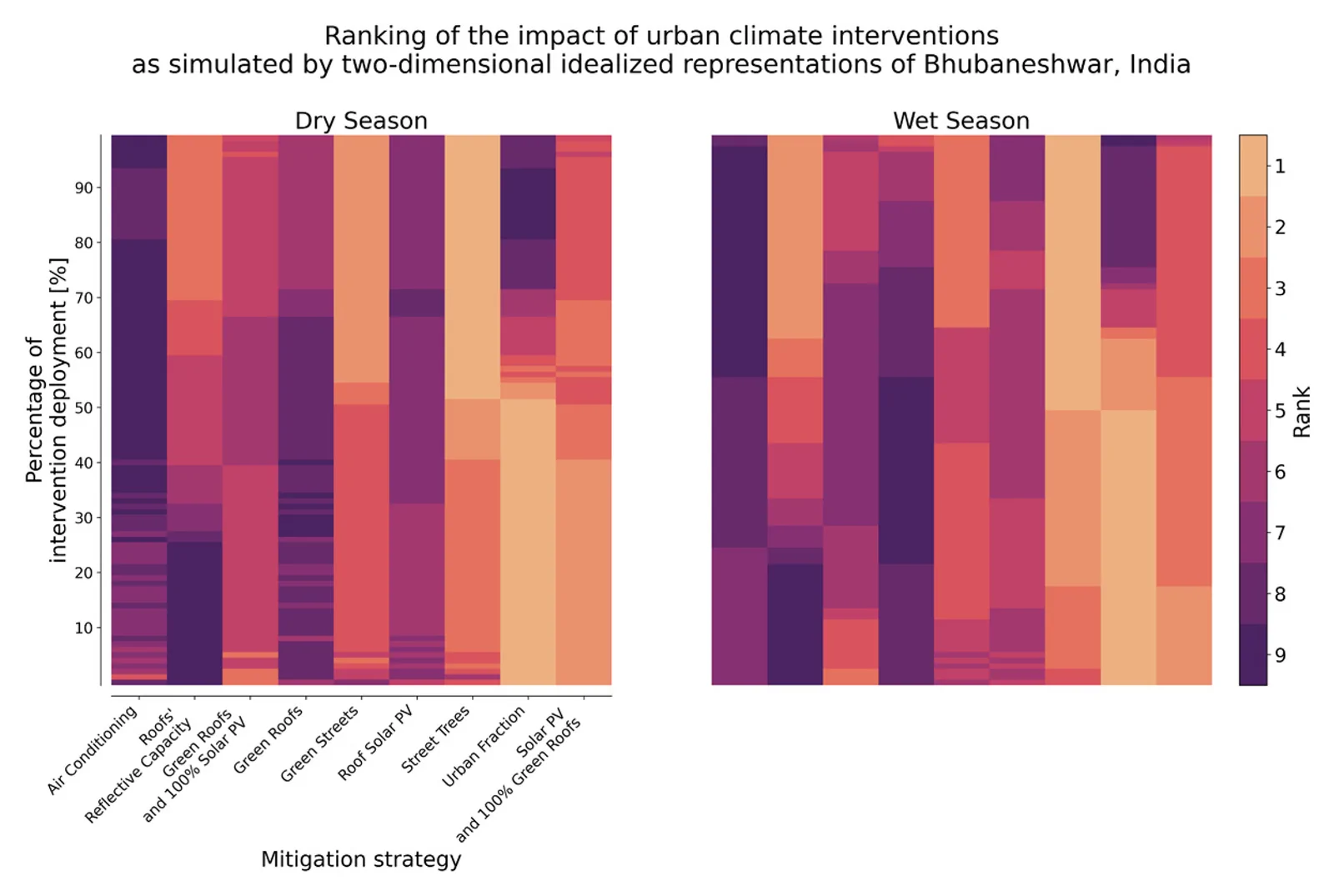
Based on idealized simulations, Street Trees systematically outperform all other interventions for average temperature reductions over the two simulated days of the dry and wet season (at 0600 IST 29–31 May 2024 and at 0600 IST 1–3 Oct 2024, respectively) when deployed at more than ~50%. Ranks are given for each intervention and for each percentage point increase in intervention deployment. Brighter colors represent better-performing interventions for daily average temperature reduction.

This exercise can be achieved for any intervention and can take forms of surface energy balance comparison or simply comparing rankings of average and maximum achievable cooling across all interventions. When looking at rankings of cooling capacities between interventions (Fig. 4), we found that in both seasons, no intervention could bring the temperatures down to equivalent temperatures observed in a city with an urban fraction of ~50%. Air conditioning is always the worst intervention as it heats up outdoor temperatures. Besides, in both seasons, Street Trees systematically outperforms other interventions in idealized simulations beyond a deployment of ~50%. Above ~70% of deployment, Street Trees is followed by Green Streets or Cool Roofs, composing a pool of three top interventions. We can also observe that there is variability in ranking of different interventions depending on the extent of their deployments. This can be better explained by looking at average changes in temperatures for each percentage point increment in deployment (Figs. 2b, 3, and Figs. S1–S19).

For instance, we find that AC Systems would contribute to increased outdoor air temperatures during the dry season days, particularly when at least ~90% of the buildings are covered by AC systems (Fig. 3). For the same days, noticeable decreases in temperatures are observed when green roofs are deployed at more than ~70% (Fig. 3), with (Green Roofs PV) or without solar PV (Green Roofs). Despite increasing albedo, Solar PV would have negligible effects on average temperature. We indeed find that the maximum average cooling is of 0.05°C of cooling. However, nighttime temperatures decrease past a deployment of ~10% (Fig. 3, Fig. S7), probably due to their lower thermal capacity compared to bare roof surfaces. Similar lack of impact by photovoltaic solar panels is also observed in Solar PV GR, where the maximum cooling of 0.42°C is similar to the maximum cooling of 0.38°C obtained in Green Roofs PV. However, while achieving this maximum cooling would require a deployment of green roofs by 99% of the roof surface under a fully covered roof by solar PV under the Green Roofs PV scenario, the maximum cooling of the Solar PV GR would be reached by deploying solar PV over 75% of a roof surface entirely covered by green roofs.

**Fig. 5:**
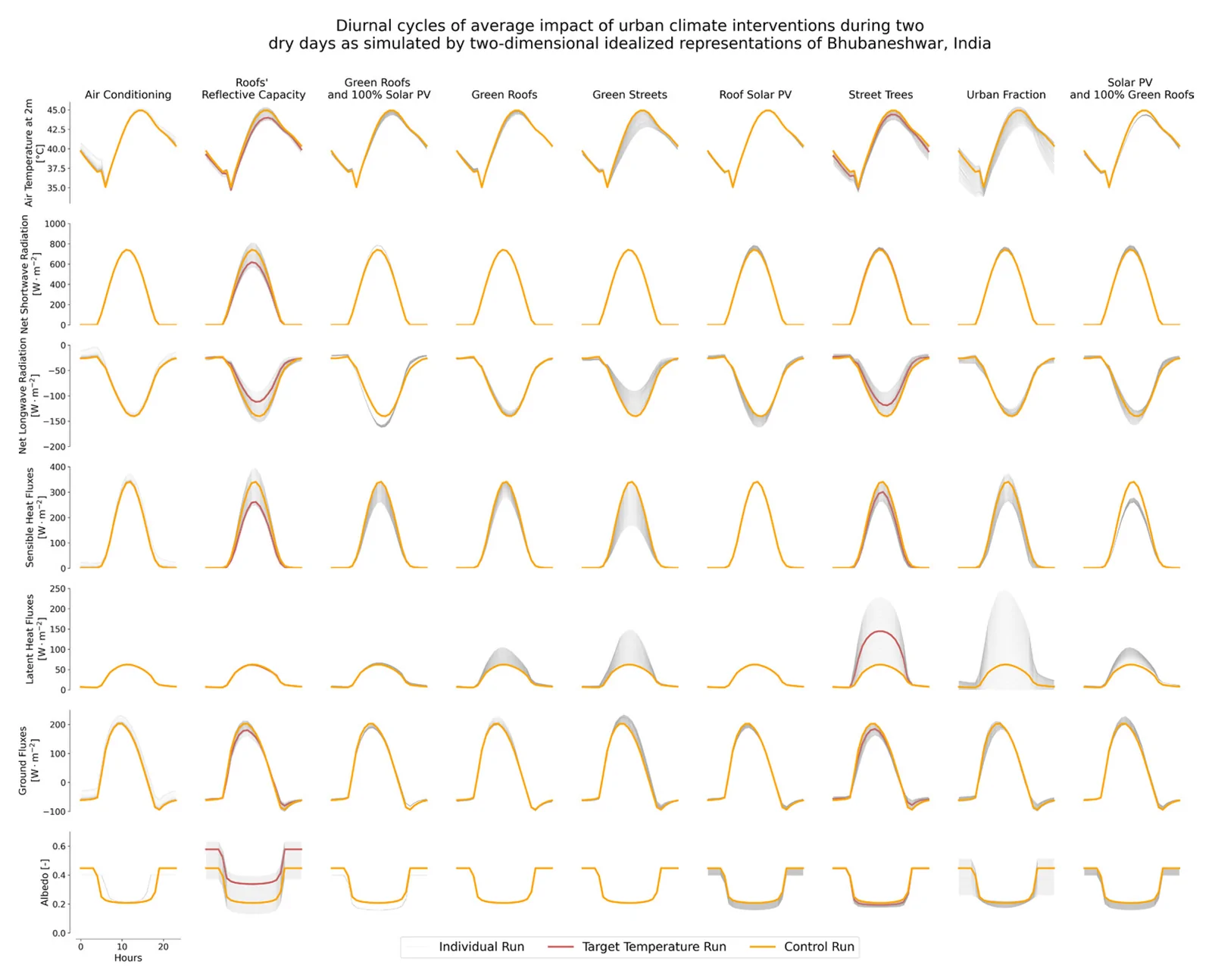
Average diurnal cycles over the 2-day simulations for each intervention per percentage point of a change (gray lines) and baseline scenario diurnal cycles (orange). Red lines in the Cool Roofs and Street Trees panels represent the expected diurnal cycle for an idealized target reduction of average temperature set at −0.5°C. Single figures of diurnal cycles and similar figures for the wet season days are provided in the S3 (Figs. S20–S38).

Inspection of the diurnal cycles of temperature and surface energy balance components (Fig. 5, Fig. S20) reveals general patterns indicating how each intervention affects the local thermal fluxes. This is important as multiple cities may set different objectives depending on their local climate conditions or on certain cobenefits to be maximized (e.g., increased biodiversity, improved water management, development of areas prone to leisure activities). In the case of Bhubaneshwar, as the local climate is very warm and humid and intense rainy events are observed during the monsoon, interventions that prevent increased levels of humidity during the dry period and that help reduce water run off may be preferred. By looking at the diurnal cycles of surface energy balance components during the dry season days (Fig. 5), one can then see that while Street Trees could lead to more than 200 W m^−2^ being emitted in the form of latent heat fluxes during hottest hours of the day, Green Streets would not exceed 150 W m^−2^, and Green Roofs would reach a maximum of ~100 W m^−2^. This means that if green infrastructure would be chosen for water management and adaptation to increasing temperatures, green roofs could be a better option if one would minimize the risk of emitting more water vapor into the atmosphere in an already highly humid environment. Additionally, clear patterns of the times at which greater impacts could be achieved by increasing the deployment level from each intervention are observable (Fig. 5). For example, Green Streets and Cool Roofs deployment would mostly reduce air temperatures during the day, while increased outdoor air temperature due to sensible heat flux from AC Systems would especially be observed at night before sunrise.

Typically, stakeholders and decision-makers may be more interested in the impact of each intervention on more easy to understand and relatable variables than surface energy balance components, such as heat indices, or changes in energy consumption/production, which can all be estimated using WRF BEP–BEM, or similar urban climate models. Researchers, on the other hand, may be more familiar with changes in the surface energy balance components, or any other land–atmosphere variable, as they would offer opportunities for developing a greater understanding of the mechanisms that drive performance and impacts of urban climate interventions. In the end, simpler simulations can quickly offer a high-level overview of the impact of different climate-sensitive design strategies at a glance, which can then guide the choice and setup of more detailed simulations that may then offer more robust estimates of the expected impacts, which can be useful for decision-makers and researchers.

## 4 Results: Comparison to a spatially realistic simulation

**Fig. 6:**
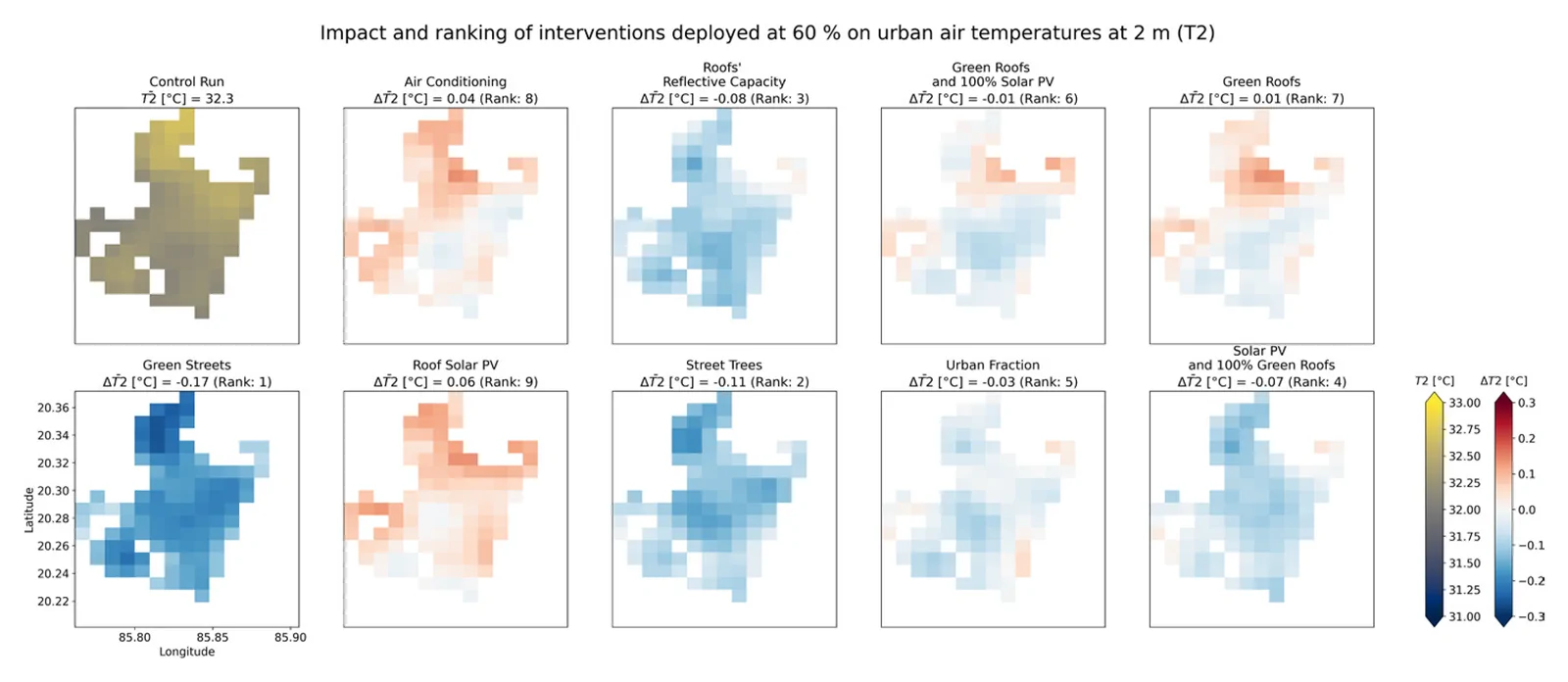
In Bhubaneshwar, Green Streets outperforms other interventions when deployed at 60% under default two-way nesting real-case scenarios during the dry season (29–31 May 2024). Average air temperatures are given for the control run in the top-left panel, while average temperature reductions induced by each interventions at 60% are provided in the remaining nine panels.

Although general principles that can help guide decision-making and expand our understanding of the impacts of interventions on the local climate can be obtained through idealized simulations, the question about their transferability remains. To try to better understand how outcomes from idealized simulations can adequately inform research or decision-making, we first investigated if ranks of performance between each intervention when deployed at 60% are similar between idealized and real-case scenarios. According to idealized simulations, at this level of deployment, interventions would be ranked in the following order according to their cooling capacities: Street Trees, Green Streets, Solar PV GR, Urban Fraction, Cool Roofs, Green Roofs PV, Solar PV, Green Roofs, and Air Conditioning (see Fig. 4). However, ranks diverge when running the model in its default real-case scenario (Fig. 6). Nevertheless, pools of better- and worse-performing interventions remain similar (e.g., Street Trees, Green Streets, Cool Roofs, and Solar PV GR). Part of this discrepancy could be explained by the spatial variability of cooling capacities observed in real-case scenarios run in their default setup. This could also explain why average temperature reductions for the dry season case are overestimated in idealized simulations compared to real-case simulations.

**Fig. 7:**
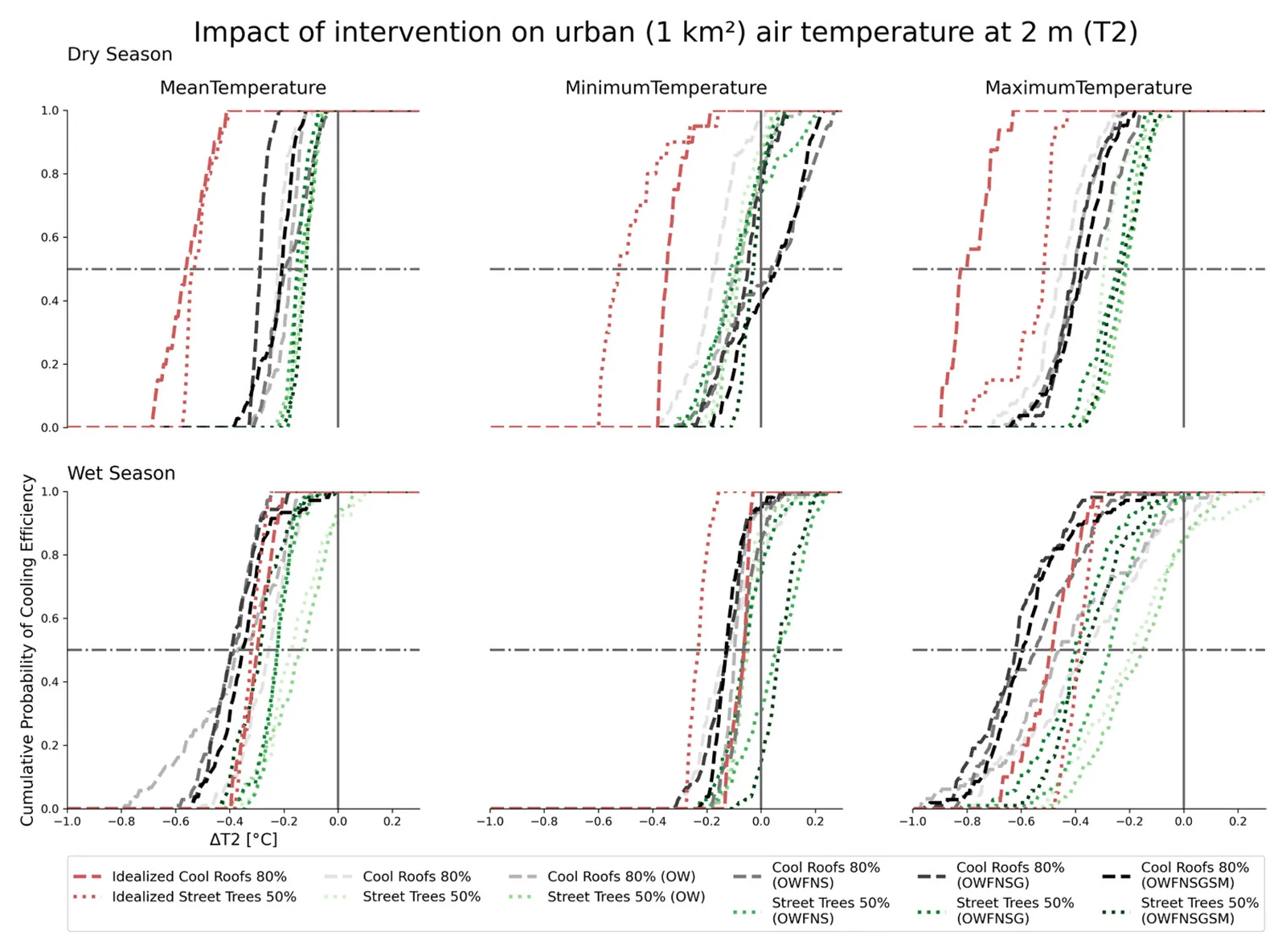
Cumulative probability plots showing the cooling capacity of the chosen interventions of cool roofs with 80% or 90% reflective capacity for dry and wet seasons, respectively, and street trees deployed at 50% under different nesting strategies (OW), with presence/absence of ocean and topography (FNS), uniform rural land cover made of grass (*G*), and uniform SM. The temperature reduction is smaller than expected in real simulations during the dry season, but street trees and cool roofs do have similar average cooling capacities.

**Fig. 8:**
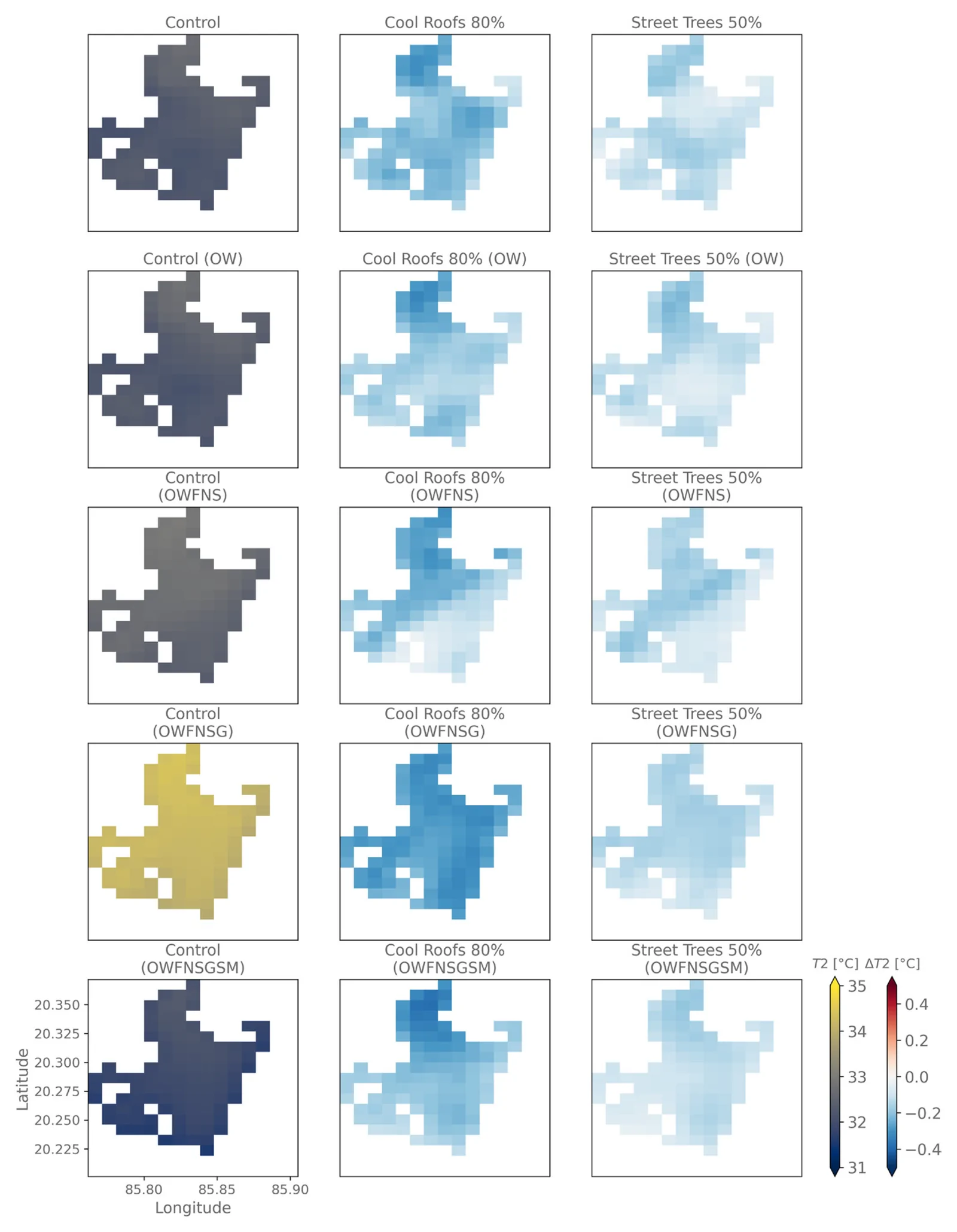
(left) Control runs over Bhubaneshwar with different nesting strategies (OW), with presence/absence of ocean and topography (FNS), uniform rural land cover made of grass (*G*), and uniform SM. (center) The related impact of cool roofs on the temperature at 2 m (T2), with an 80% reflective capacity; (right) street trees covering 50% of the streets. The more the model resembles the idealized runs, the more values converge toward a uniform spatial pattern, similar to the idealized simulations. A similar figure for the wet season’s days with cool roofs set at 90% reflective capacity and street trees covering 50% of the streets is given in the S3 (Fig. S39).

We therefore looked at the simulated temperature reduction brought by Cool Roofs at 80% and 90% for dry and wet seasons, respectively, and Street Trees at 50%, for both seasons, in more realistic case scenarios. Both levels of interventions were expected to lead to temperature reductions close to the target temperature of −0.5°C for the dry season and of −0.3°C for the wet season, based on idealized scenarios. Interestingly, we see that when running the WRF BEP–BEM in realistic mode, for the same two warm days, and over a realistic city of Bhubaneshwar parameterized in the same way as the city archetype defined in the idealized simulations, temperature reductions are lower in magnitude in both seasons (Fig. 7). It therefore appears that the idealized simulations tend to overes-timate the cooling capacity of the chosen interventions in our case study. In fact, Cool Roofs at 80% reflective capacity during the dry season led to an average temperature reduction of ~0.3°C, while Street Trees at 50% decreased temperatures by ~0.2°C on average (Fig. 7). However, during the wet season, the cooling brought by Cool Roofs at 90% reflective capacity is closer to the expected temperature reduction of ~0.3°C, while the Street Trees lead to an average temperature reduction of ~0.2°C. Such misalignments could be expected because of the added complexity in the land, sea, and atmospheric interactions that are induced by running the WRF Model in real conditions. On top of this, idealized simulations are simply launched from initial conditions with a constant wind speed and orientation, while real scenarios require forcing at the boundaries every few (usually every six) hours, and are two-way nested, meaning inner domains inform outer domains about the simulated local climate. In the case of the dry season, this could lead to amplifications of air temperatures that are not seen in the real-case scenario, and which could explain the greater cooling capacity overall. Therefore, when running real-case scenarios that are more proximate to idealized simulations in their parameterization, we would expect to see more relatable patterns (Figs. 7 and 8, and Fig. S39).

We find noticeable changes in the cooling capacity induced by the parameterization of more “idealized conditions” (in our case: one-way nesting with flat terrain, no sea, and only grass land in surrounding rural areas with equal soil moisture; OWFNSGSM); the cooling capacity remains lower than in the idealized simulations (Fig. 7), especially during dry season. In fact, we find that for both wet and dry season cases (Fig. 8 and Fig. S39), running more “idealized” real-case scenarios notably changed the cooling capacity and made them behave more similarly to idealized cases. For instance, outputs for the cool roofs scenarios with the OWFNSG case are closest to the expected 0.5°C of average cooling (Fig. 8), while both cool roofs and street trees scenarios during the wet season converge to the expected 0.3°C of average cooling and observe smaller relative differences (Fig. S39). Indeed, when looking at maps of temperature reductions in the real-case scenarios (Fig. 8), more uniform spatial patterns in cooling potential arise by moving toward the idealized parameterization. Noteworthy, the last step of uniform soil moisture did not yield more cooling than the uniform grass land cover in the rural areas (Fig. 8).

In short, on average, changes in temperatures are similar between Street Trees and Cool Roofs interventions, despite the lower cooling capacity for real-case scenarios in the dry season. This demonstrates that the physical principles underlying the idealized simulations hold for the real-case scenarios despite a different magnitude in the impact. This also further suggests that to expect real-case scenarios to achieve similar temperature reductions, initial conditions in the land and the atmosphere should be similar to the idealized scenario.

## 5 Discussion

In this study, we demonstrated how current practices in urban climate modeling do not respond to the climate urgency that cities face and how idealized simulations set up in the WRF Model could help to provide valuable information to support action against extreme heat in urban areas. Through the example of Indian cities—and more specifically Bhubaneshwar, located in the state of Odisha, on the eastern coastal belt of India—we developed a framework that could be foreseen as central to any urban climate impact studies. In Bhubaneswar, and elsewhere in India, mortality and morbidity associated with heat stress such as those experienced in 2024 is a serious and persistent concern, already observed in previous years (e.g., de Bont et al. 2024). In spite of the existence of city-level heat action plans such as those for Bhubaneswar (Odisha State Disaster Management Authority 2026), heat governance continues to pose challenges.

To help define future pathways in the building of resilient cities with decision-makers, our framework enables a rapid expansion of our understanding of the impact of urban interventions on the local climate while relying solely on openly accessible tools and global data: the Weather Research Forecasting Model (Chen et al. 2011), global local climate zones maps (Demuzere et al. 2022b), and ERA5 climate reanalysis data (Hersbach et al. 2020). Using this framework, we show how two-dimensional idealized simulations could efficiently help scoping potential viable options for reducing negative urban climate impacts, such as overheating. While running a real-case scenario may involve several hours of computational time, in this case ~ 8 h 45 per simulation requiring 200 processors, running an idealized simulation on a single processor took 2–3 h. The latter means that for the same amount of processors, 200 idealized simulations could be performed in ~3–4 times less time compared to one single real-case scenario. Obviously, this depends on each machine’s specifications and computing infrastructures. But, this shows the extent of scalability of idealized simulations to address simple questions on the expected impacts of climate-sensitive designs and/or adaptative or mitigative solutions, useful to decision-makers. Until now, idealized simulations have not been proposed as a tool for scoping for viable urban climate interventions. Previous studies from Pappaccogli et al. (2020) or Zonato et al. (2021), for example, already employed idealized simulations to perform sensitivity analyses of changes to model inputs. Besides, Martilli (2014) developed idealized simulations of hypothetical square-shaped cities to test how city structures influenced by different realistic population densities influence energy consumptions, air temperatures, and air pollutions. Here, we demonstrate how simple idealized simulations of real-case scenarios that avoid the physical and computational complexities of three-dimensional simulations can be used to inform the parameterization of more complex simulations.

We therefore argue that simpler simulations should be used before running complex simulations that, for now, rely on arbitrary choices. In fact, such rapid and comprehensive insights brought by idealized simulations would support decision-making by providing research-based evidence on the expected impacts of each intervention and what could be the related costs and benefits from each of them. This could be useful for designing local policies and foster research-based urban planning actions. Thus, our proposed model-based simulations could provide timely and meaningful contributions to authorities who are seeking solutions. In particular, this analysis adds value in all similar contexts where observational data at local level are scarce and typically resource-constrained city governments (Ahluwalia 2019) are seeking to prioritize interventions that are likely to be most effective in managing heat such as through heat risk informed urban planning (Dasgupta et al. 2025). Evaluating how useful this framework is to decision-making will nonetheless have to be resolved in the future, depending on the uptake of our proposed strategy.

Notably, we showed how idealized simulations can quickly represent the impact of numerous potential climate interventions on the local climate in an easily understood manner. They also offer intuitive details on how these impacts are expected to arise, exemplified through the surface energy balance changes. These are key strengths of our suggested framework. Whether these impacts are accurate or not is not the purpose of this work—although this remains a key limitation. In fact, many uncertainties arise from the parameterization stages to the difficulties arising in the model evaluation against measurements that would not be representative of such idealized conditions. Instead, this framework is supposed to provide physically sound material that can help discussing which climate intervention is the most appropriate to local urban decision-makers. This can depend on a single objective, like air temperature reduction, or multiple objectives like thermal comfort and rainfall management. By comparing outputs from realistic and idealized simulations, we actually showed that the idealized simulations do offer reliable physical environments, similar to more complex and realistic models. This allows for a valid approximate comparison of the local climate impact from different climate interventions that can be further extended to studying cobenefits between interventions.

For Bhubaneshwar, we demonstrated how idealized simulations can help in understanding complex phenomenon in urban climate intervention impacts, such as the (non)linear sensitivity of simulated local climate impacts to changes in interventions’ deployment. Because (non) linear sensitivity of urban climate models to changes in urban climate interventions remains poorly explored, this could translate to a lack of awareness of the scales of deployment required to achieve optimal adaptation to climate changes—exemplified through air temperature reduction and surface energy balance alteration. This could also suggest that previous studies that tried to model the impact of urban climate interventions through linear regressions only may have high levels of uncertainty. Additionally, common urban climate modeling practices that study single or few intervention extensions [e.g., studies listed in Krayenhoff et al. (2021) or Santamouris et al. (2017)] will not offer insights on intermediate extensions that can lead to similar impacts. Such information could be central to decision-making processes as this directly relates to deployment and maintenance costs. Last, nonlinearity again reflected how simple simulations can support a better physical understanding of climate interventions, relevant to decision-making at early stages.

In this study, we decided to oversee the whole range of application of idealized simulations by running sets of 100 simulations for each intervention percentage change. Possibly, smaller sets of simulations with regressions trained over their outputs to fill the gaps could be considered in the future—depending on the computational capacities of people engaging with simpler simulations. Yet, this would require a better understanding of the sensitivity of dynamic models to different urban environments embedded in multiple climatic conditions to guide users in the minimum number of simulations that need to be run. The latter is out of the scope of this study and will need future work to be achieved.

Our work suffers from some limitations. First, we were not capable of gathering air temperature and radiative measurements to validate our model outputs for idealized simulations. We argue that, for cases like Bhubaneshwar in India and other rapidly growing cities, this should not prevent the use of simple simulations as a guiding tool for research design and decision-making, especially considering the urgency in face of climate actions. On top of this, idealized representations of a city should be as realistic as possible in their representations of the studied city, but we do not expect model performance of similar quality to that achieved in real-case scenarios as idealized simulations are not regularly forced by lateral boundary conditions. Deployment of urban weather networks from official and crowdsourced sources should continue to be encouraged to address this limitation as soon as possible. Second, our model of the fractional air conditioning impact on urban climate remains simplistic and is only intended to produce city average sensible heat fluxes that depend on fractional intakes of AC across the urban environment. More realistic representations that split estimations of sensible heat fluxes emissions from indoor built-up environments to outdoors between buildings with and without AC could be developed in the future, similar to Lipson et al. (2019). Tiling approaches that would separate between land-use and land-cover urban classes that compose each urban grid could also be envisioned. Yet, both these solutions would induce exponential computational costs, especially under real-case scenarios; exactly what our frame-work is designed to negate. Last, all presented impacts of interventions in this manuscript should be cautiously interpreted as they are dependent on short simulations with specific parameterizations [e.g., green roofs are not irrigated, which could decrease their cooling capacity; see Zonato et al. (2021)].

It is also important to highlight that some limitations exist in the current design of the idealized simulations framework. For instance, we found that the transferability of the idealized outputs to real-case scenarios was not straightforward. In fact, specific attention to the similarity of the idealized simulation to the real-case environment should be paid as the modeled impact of local interventions seems to be greatly dependent on initial and boundary model conditions. This remains a challenge that will require further research as many parameters change between an idealized simulation and a real-case scenario, despite efforts to keep physical and dynamical parameterizations identical. Besides, real-case simulations should not be disregarded nor abandoned from impact research processes. However, instead of being seen as a first step toward decision-making and research actions, they should really be used as a way for confirming hypotheses gathered during idealized simulation phases and/or as valid tools for representing real-world phenomena. Representing a city with 100% coverage of green roofs remains an “ideal” which offers little more information than having chosen to represent the city with 80% green roofs coverage instead if the intermediate impacts remain unknown. After all, models are always idealized representations of the real world.

## 6 Recommendations for future users

Based on the outcome of our study and the limitations identified in the discussion, we provide a list of recommendations for future users:

1) Idealized simulations should represent the studied cities and climates in the most realistic way as practical. As shown over Bhubaneshwar, our idealized simulations were parameterized in order to model an archetypical city with representative morphologies of a similar size to the actual city. In addition, initial conditions including soil moisture content, temperature, and lateral boundary conditions were all derived from climate re-analysis data. This is mandatory and ensures that the studied impacts are not random.
2) Outputs from idealized simulations should always be carefully interpreted and not considered final. Users should systematically use this framework for exploration of potential interventions rather than the study of the expected impact under real conditions. In particular, this framework is meant to help quickly understanding which interventions are better than others for different cities under distinct climate conditions.
3) Once potential interventions are identified, real-case scenarios should be run to estimate the actual impact of interventions on the city. Idealized simulations cannot be used on their own for estimating the impact of interventions. It is necessary that this final step is systematically undertaken to adequately inform decision-makers about the impact the interventions may have.
4) When possible, real-case control runs should be evaluated against official and/or crowd-sourced weather records. This is still the only way for adequately communicating about measured model’s uncertainties. Importantly, model’s uncertainties for control runs are not necessarily transferrable to modeled impacts of interventions. Therefore, if modeled impacts of interventions are of lower magnitudes than quantified model errors, then the modeled impacts should be considered uncertain (Krayenhoff et al. 2021). Globally, records of impacts of interventions are missing and prevent the evaluation of model’s uncertainties.
5) Uncertainties and limitations of the framework should be presented to all parties involved in decision-making. It is the duty of users of this framework to be transparent about what is actually being tested and how. Again, this framework is only meant to support initial discussions by providing tangible data for deciding which intervention may be the most suitable for each city. This should be clearly enunciated at the start of each project and whenever this framework is employed.

## 7 Conclusions

Through the example of the city of Bhubaneshwar in the state of Odisha, India, we present an innovative framework aimed at supporting early decision-making for urban climate intervention. Our framework relies on the employment of two-dimensional simplified simulations, which are shown to efficiently represent the wide range of impacts of common urban interventions, tested for two warm days of the dry and wet seasons of 2024 in Bhubaneshwar. Two-dimensional simulations should always be complemented by real-case modeled scenarios to adequately inform about the expected impact of the chosen interventions.

## Supplementary Material

Supplemental information related to this paper is available at the Journals Online website: https://doi.org/10.1175/BAMS-D-25-0197.s1.

## Data Availability

Adapted models, input data, and codes for data analysis are all available in a dedicated Zenodo directory with https://doi.org/10.5281/zenodo.21074194.

## Significance Statement

Scoping for viable solutions to respond to challenges imposed by global climate changes and local urban climates on urban populations can be difficult. Current modeling practices involve climate models that are complex and use a lot of resources, therefore limiting the amount of “what-if” scenarios that can be run. We hereby propose a framework that employs simplified simulations to inform about viable interventions at the city scale to help design responses to local climate challenges in the most adequate and efficient way. Our study focuses on the city of Bhubaneshwar in the state of Odisha, India, and shows the applicability of this framework in areas that are understudied and critically impacted by climate changes.
